# High-Performance Quasi-2D Perovskite Light-Emitting Diodes Via Poly(vinylpyrrolidone) Treatment

**DOI:** 10.1186/s11671-020-3260-z

**Published:** 2020-02-04

**Authors:** Zijun Wang, Xiaoqiang Xu, Lin Gao, Xingwu Yan, Lu Li, Junsheng Yu

**Affiliations:** 1 Research Institute for New Materials Technology, Chongqing University of Arts and Sciences, Chongqing, 402160 People’s Republic of China; 2 State Key Laboratory of Electronic Thin Films and Integrated Devices, School of Optoelectronic Science and Engineering, University of Electronic Science and Technology of China (UESTC), Chengdu, 610054 People’s Republic of China

**Keywords:** Quasi-2D perovskite, Perovskite light-emitting diodes, PVP, Morphology control, Repeatability

## Abstract

In this work, we fabricate poly(vinylpyrrolidone) (PVP)-treated Ruddlesden-Popper two-dimensional (quasi-2D) PPA_2_(CsPbBr_3_)_2_PbBr_4_ perovskite light-emitting diodes (PeLEDs) and achieved a peak brightness of 10,700 cd m^−2^ and peak current efficiency of 11.68 cd A^−1^, threefold and tenfold higher than that of the pristine device (without PVP), respectively. It can be attributed that the additive of PVP can suppress the pinholes of perovskite films owing to the excellent film-forming property, inhibiting the leakage current. Besides, PVP treatment facilitates the formation of compact perovskite films with defect reduction. Our work paves a novel way for the morphology modulation of quasi-2D perovskite films.

## Introduction

Perovskite light-emitting diodes (PeLEDs) have aroused significant attention for application in next-generation electroluminescence due to their high photoluminescence quantum yield (PLQY), tunable bandgap, high color purity, and great charge transport properties of metal halide perovskites [1–10]. In just 5 years, efficiency advance for PeLED has been made from < 1 to > 20% [1, 4, 5]. At first, organic-inorganic hybrid perovskites (OHIP), such as MAPbBr_3_, have been employed widely as an emitting layer in the fabrication of PeLEDs [2, 11–13]. However, they have been replaced gradually by all-inorganic perovskites, such as CsPbBr_3_, since chemical and thermal stabilities of OHIP are subject to debate for the weak binding force between their organic cations and metal anions [14, 15].

It should be noted that when pure CsPbBr_3_ is used as an emitter in PeLEDs, the performance is often hindered due to the severe current leakage and high non-radiative recombination, caused by low surface coverage and grain boundary defects [16–18]. Besides, small exciton binding energy of 3D (bulk) perovskites at room temperature will result in low PLQY at low excitation intensity, unfavorable for the performance of resultant PeLEDs [19–21]. Hence, Ruddlesden-Popper two-dimensional (quasi-2D) perovskites, generally known as *L*_2_(CsPbBr_3_)_*n*-1_PbBr_4_ with layered structures have become hot research materials in PeLEDs, where *L* and *n* represent long-chain alkyl or phenyl group and the number of PbBr_4_ octahedral layers within a crystallite, respectively. The introduced *L* actions cannot fill into the interspace of [PbBr_6_]^4−^ octahedral because of large ionic radius, resulting in the formation of layered perovskite film with self-assembly multiple quantum wells (MQWs) structure via spin coating, which is a mixture of layered perovskites with different *n* numbers and different bandgap [22]. For instance, organic ammonium salts such as phenethylammonium bromide (PEABr) [23, 24], butylammonium bromide (BABr) [25, 26], phenylbutylammonium bromide (PBABr) [27], and propylammonium bromide (PABr) [28] have been incorporated with CsPbBr_3_ to form quasi-2D perovskites. Ng et al. employed PEABr as a long-chain group cooperating with CsPbBr_3_ in the fabrication of quasi-2D PeLEDs. The current efficiency (CE) has been improved to 6.16 cd A^−1^ since the efficient energy funneling and morphological control [24]. Wang et al. demonstrated high-performance quasi-2D PeLEDs-based BA_2_(CsPbBr3)_*n*-1_PbBr_4_. The maximum luminance of PeLEDs is dramatically enhanced from 191 to 33,533 cd m^−2^ through polymer doping and solvent treatment compared with the 3D CsPbBr3 devices [25]. Chen et al. reported a high-quality quasi-2D perovskite film of PA_2_(CsPbBr3)_*n*-1_PbBr_4_ with highly dense, smooth morphology, and a high PLQY, which is used as an emitting layer in the fabrication of blue PeLEDs with a maximal external quantum efficiency (EQE) of 3.6% [28]. Due to the efficient energy funneling from larger bandgap (2D) domains to the lowest bandgap (3D) radiative domains performance in quasi-2D perovskites, these materials can promote the radiative recombination as well as higher PLQYs [20]. It is beneficial for obtaining high-performance PeLEDs. Meanwhile, the large organic bulky cations can facilitate the formation of compact perovskite films. Therefore, the quasi-2D perovskite film exhibits high coverage and low roughness due to the inclusion of large organic cation [29].

Hence, in our previous work, long-chain ammonium cation (phenylpropylammonium (PPA)) was introduced, enabling the formation of PPA_2_(CsPbBr_3_)_2_PbBr_4_ for quasi-2D PeLEDs through adjusting Cs ratio [30]. However, since there exist lots of pinholes in perovskite films, which cause serious leakage current, the performance of quasi-2D PeLEDs we reported still needs to be further improved for meeting the actual application. Besides, this pinhole phenomenon not only occurs in our previous report but also others’ report about quasi-2D perovskite-based CsPbBr_3_ [24, 31]. It is necessary to find a method to solve the pinhole problem in fabricating perovskite films for improving device performance.

In this study, a wide applied polymer, poly(vinylpyrrolidone) (PVP) [32], with moderate electrical conductivity and the excellent film-forming property was first introduced as an additive to control the morphology of quasi-2D CsPbBr_3_ perovskite films for fabricating the PeLEDs with high luminance and CE. Adopting appropriate ratio, PVP can improve the compactness of perovskite films while ensuring the smaller grain size, reduce grain boundary defects, and suppress the pinholes. Hence, smooth and pinhole-free quasi-2D perovskite films are demonstrated with suppressed current leakage and non-radiative recombination losses, which greatly improves the luminance and efficiency of PeLEDs. The best PeLED yields a maximum luminance and CE of 10,700 cd m^−2^ and 11.68 cd A^−1^, respectively, threefold and tenfold higher than that of the pristine device (without PVP), respectively.

## Methods

PbBr_2_ (99.999%), CsBr (99.999%), poly(vinylpyrrolidone) (PVP), and LiF were purchased from Sigma-Aldrich. Dimethyl sulfoxide (DMSO) was purchased from Alfa Aesar. Poly(3,4-ethylenedioxythiophene):poly(styrene-sulfonate) (PEDOT:PSS) (AI4083, Heraeus), 1,3,5-tris(2-*N*-phenylbenzimidazolyl) benzene (TPBi), and PPABr were purchased from Xi’an Polymer Light Technology Corp. All the materials were received without further purification.

**Fig. 1:**
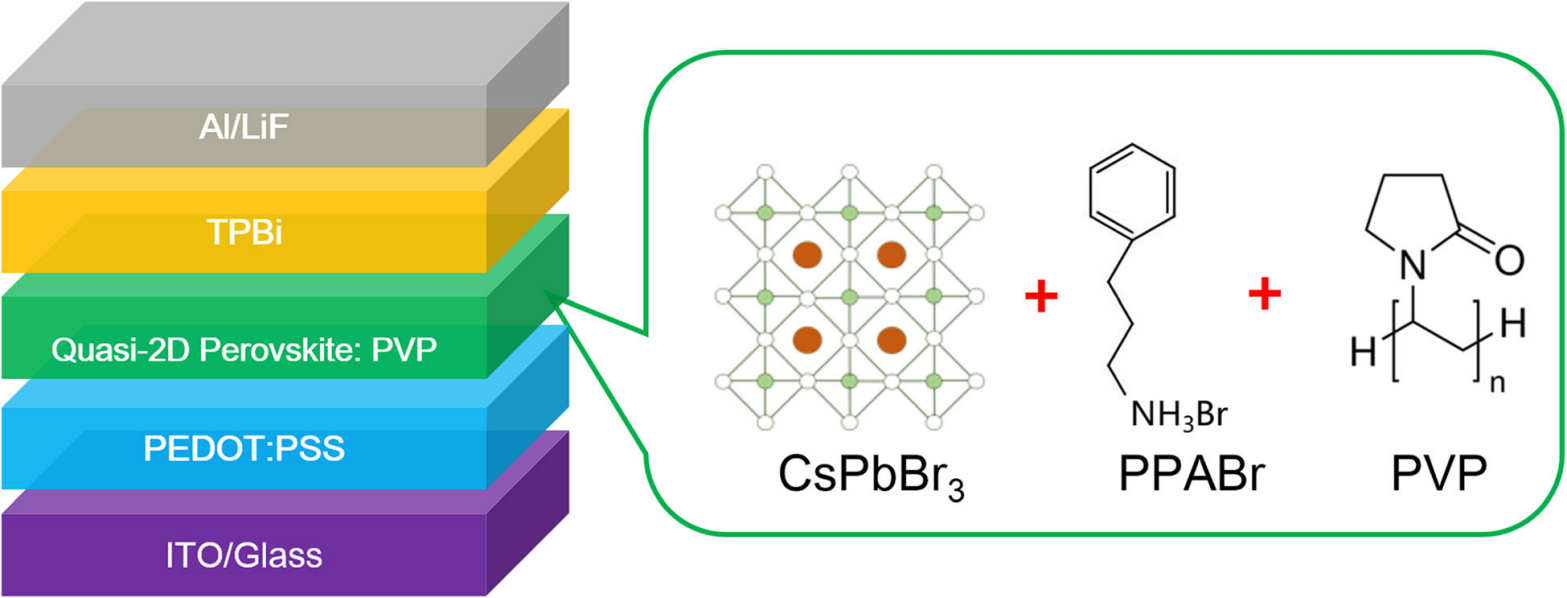
Device architecture of quasi-2D PeLEDs and the chemical structure of the emitting layer

The quasi-2D PeLEDs were fabricated with the structure of indium tin oxide (ITO)/PEDOT:PSS/quasi-2D perovskite with or without PVP/TPBi/LiF/Al as shown in Fig. 1. The ITO substrates were cleaned in an ultrasonic bath with detergent water, acetone, deionized water, and isopropyl alcohol, successively. Before use, the substrates were treated with ultraviolet ozone for 15 min after drying in an oven. For preparing the perovskite precursor, PVP was dissolved in DMSO with different concentrations of 0 mg/mL, 2 mg/mL, 3 mg/mL, and 4 mg/mL. All the solutions were stirred with 600 rpm at 60 °C for 6 h. Then, perovskite precursor solutions were prepared by dissolving 31.9 mg PPABr, 21.2 mg CsBr, and 55.5 mg PbBr_2_ in 1 mL above PVP-DMSO solution with different concentrations of 0 mg/mL, 2 mg/mL, 3 mg/mL, and 4 mg/mL, respectively. Then, all the perovskite solution was stirred with 400 rpm at 60 °C for 12 h. PEDOT:PSS was spin coated on the ITO substrates at 3000 rpm for 60 s to make a layer with a thickness of ~ 40 nm. After annealing at 140 °C for 20 min in air, the substrates were transferred to the glovebox filled with nitrogen for preparing perovskite lay. The perovskite films were deposited on the substrates by spin coating the precursor solutions with different compositions of PVP at 3000 rpm for 120 s and annealing at 100 for 15 min. Next, a 40-nm-thick TPBi was evaporated to cover the perovskite film, followed by the deposition of LiF (1 nm) and Al (100 nm) through thermal deposition in high vacuum condition. The overlap between ITO and Al electrodes was 0.1 cm^2^, which is the active emissive area of the devices.

All the PeLED measurements were carried out at room temperature in a nitrogen-filled glovebox. The current density-voltage-luminance (J-V-L) characteristics were collected via two computer-controlled Keithley 2400 digital source meter coupled with a calibrated Si photodiode. The morphologies of perovskite were characterized by scanning electron microscopy (SEM, ZEISS GeminiSEM 300) and atomic force microscope (AFM, AFM 5500, Agilent, Tapping Mode). The X-ray diffraction (XRD X’Pert PRO, PANalytical) measurements were adopted based on ITO/PEDOT:PSS/quasi-2D perovskite with a Cu Kα radiation source set to 30 kV and 20 mA. Absorption spectra of perovskite films on quartz glasses were measured using a Cary 5000 UV-Vis-NIR system (Agilent). The steady-state photoluminescence (PL) spectra were measured by a fluorescence spectrophotometer (F7000, HiTACHI) with a 400-W xenon lamp as the excitation source and an excitation wavelength of 350 nm. Time-resolved PL (TRPL) measurements were conducted using a fluorescence spectrophotometer coupled with a time-correlated single-photon counting (TCSPC) system.

## Result and Discussions

**Fig. 2:**
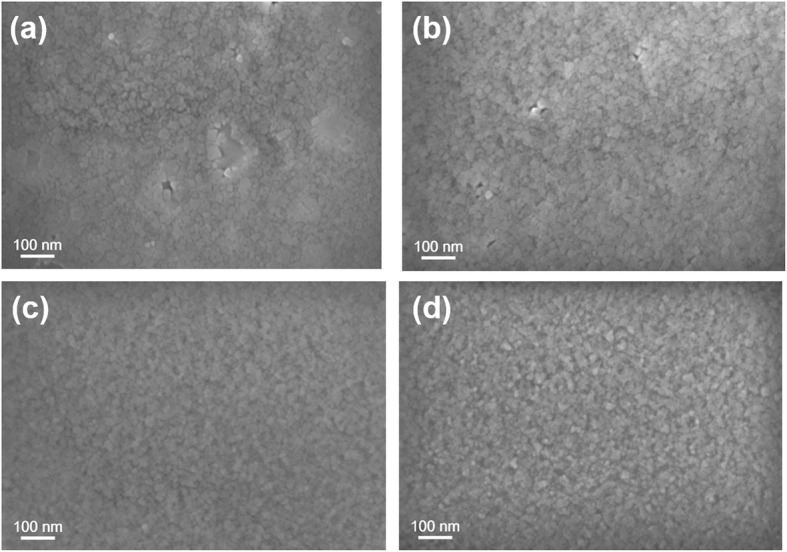
SEM images of perovskite films with **a** pure PPA_2_(CsPbBr_3_)_2_PbBr_4_ and PVP-treated perovskite with concentration of **b** 2 mg/mL, **c** 3 mg/mL, and **d** 4 mg/mL

**Fig. 3:**
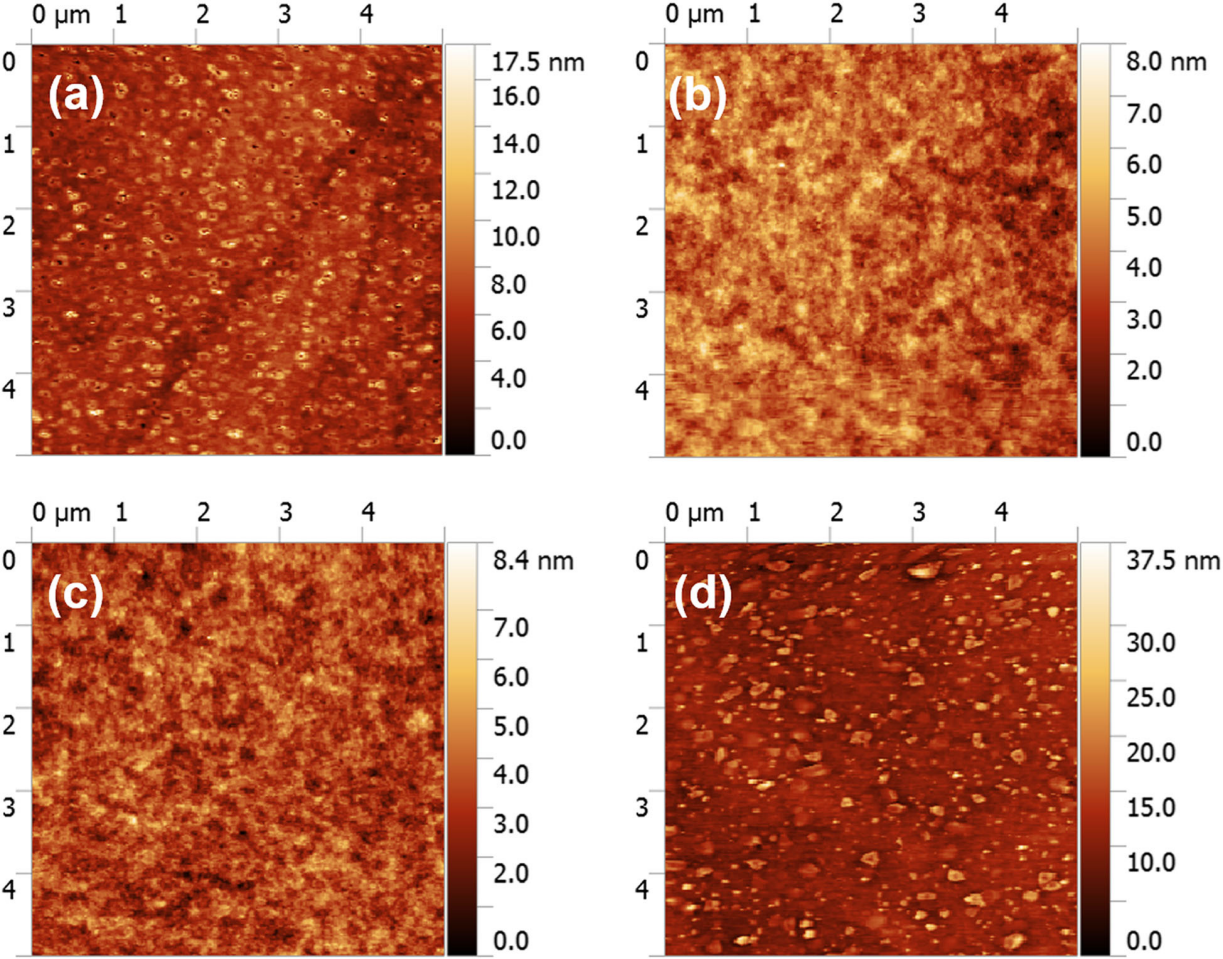
AFM topographies of corresponding perovskite films with **a** pure PPA_2_(CsPbBr_3_)_2_PbBr_4_ and PVP-treated perovskite with concentration of **b** 2 mg/mL, **c** 3 mg/mL, and **d** 4 mg/mL

The effect of PVP treatment on the morphology and crystallization of quasi-2D perovskite is first explored by SEM and AFM measurements as shown in Figs. 2 and 3. All the quasi-2D perovskite samples show complete coverage. However, as we can see from Fig. 2a and Fig. 3a, there exists a mass of pinholes in pure PPA_2_(CsPbBr_3_)_2_PbBr_4_ film with relatively large grains (≈ 30 nm) which could cause severe current leakage and then limit the device performance. According to the previous reports, the quality of perovskite films can be improved by the incorporation of polymers [10, 30]. Indeed, according to Fig. 2b–d, the morphology of perovskite has been greatly improved with the addition of PVP, exhibiting compact morphology with few pinholes. It is evident in Fig. 2b that the 2-mg/mL PVP additive enables the growth of small grains and compact morphology with few pinholes. With the concentration of PVP increase, pinhole-free perovskite film is formed as shown in Fig. 2c, d with small grains (< 10 nm). Besides, the RMS of the pure PPA_2_(CsPbBr_3_)_2_PbBr_4_ film is 1.44 nm, which is greatly decreased to 0.76 nm after the incorporation of PVP (2 mg/mL) as shown in Fig. 3a, b. With increasing the concentration of PVP to 3 mg/mL, the roughness barely changes. However, when the concentration of PVP is raised to 4 mg/mL, the surface became rough again as shown in Fig. 3d, which may be caused by the aggregation of PVP. It is unfavorable for carrier injection from the electron transport layer (ETL) to the perovskite layer. Hence, we do not further increase the concentration of PVP. The results indicate that the proper addition of PVP is beneficial for the formation of a dense, smooth, and pinhole-free perovskite film with uniform grain size.

**Fig. 4:**
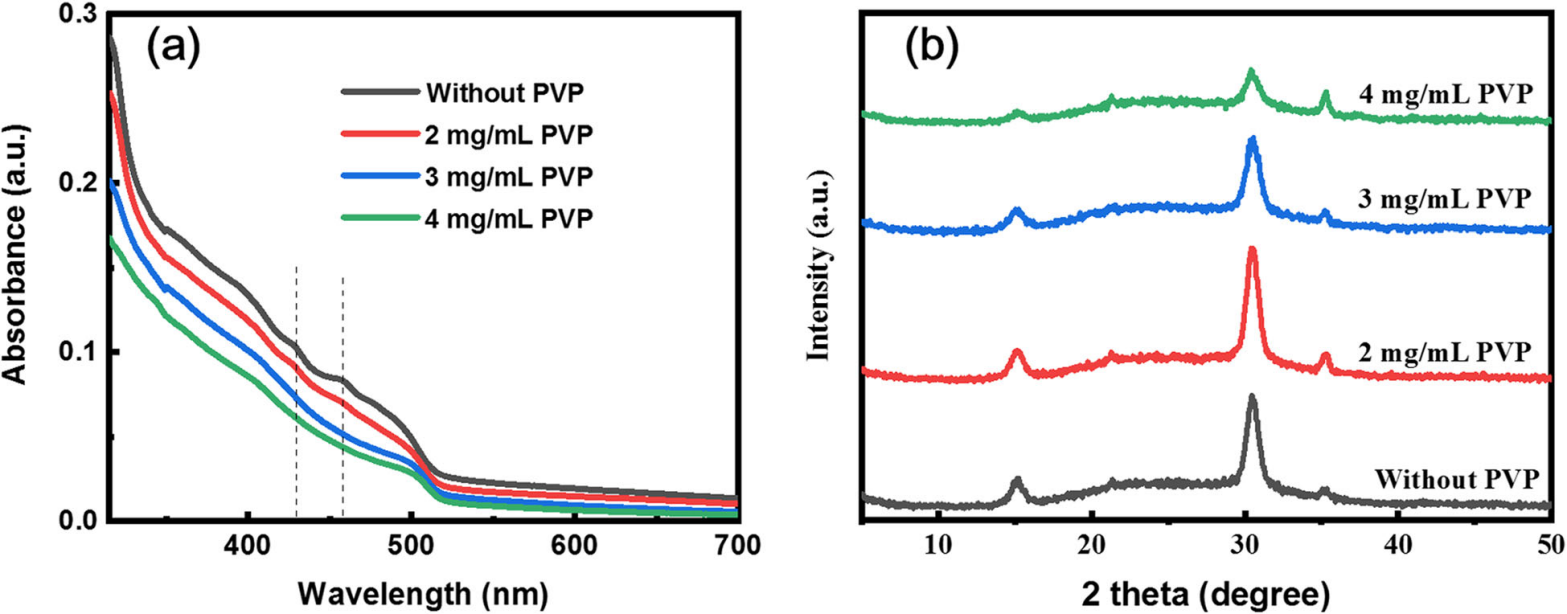
**a** UV-Vis absorption of quasi-2D perovskite films. **b** XRD patterns of quasi-2D perovskite films

The UV-visible absorption spectra of quasi-2D perovskite film were collected as shown in Fig. 4a to confirm the existence of lower-dimensional phases. The quasi-2D perovskite film without PVP as an additive has weak exciton absorption peaks at 438 nm and 458 nm, corresponding to *n* = 2 and *n* = 3 phase perovskite, respectively [31]. However, when PVP is introduced, both the exciton absorption peaks become weaker. It means that the incorporation of PVP could take the edge off the growth of small *n* value perovskite phase in perovskite film, instead of promoting the large *n* value perovskite phase. To study the influence of the incorporation of different PVP concentrations on the crystal structure of quasi-2D perovskites, XRD was conducted as shown in Fig. 4b. All perovskite films have diffraction peaks of 15.2° and 30.4°, corresponding to the diffraction peaks of (100) and (200), respectively. These observations match the cubic perovskite crystal structure, which is consistent with previous reports [33]. Besides, with the gradual increase of PVP concentration, the full width at half maximum of the diffraction peak corresponding to the (200) crystal plane becomes larger. It indicates that the growth of perovskite crystals is gradually inhibited as the PVP amount increases, which is consistent with the above SEM characterization.

**Fig. 5:**
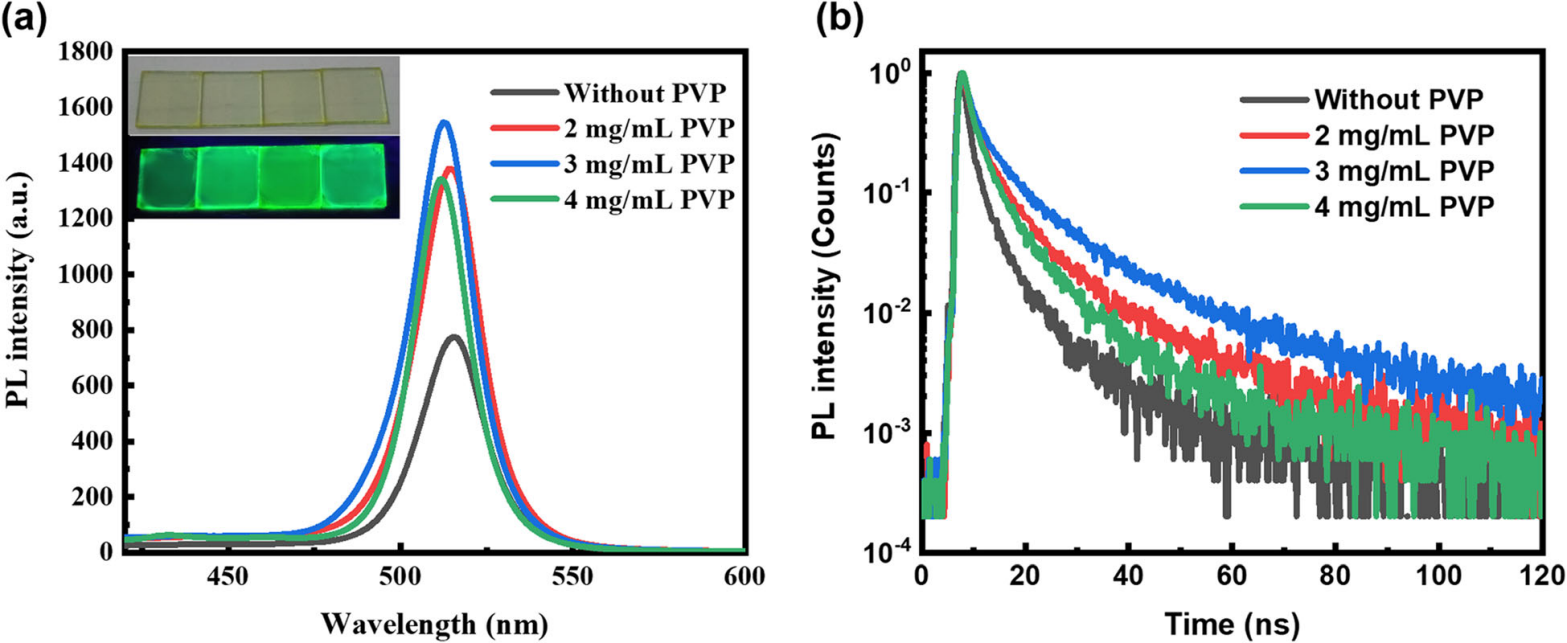
**a** PL spectra of quasi-2D perovskite films with different PVP concentrations; the inset shows the image of quasi-2D perovskite films under a 365-nm wavelength ultraviolet lamp. **b** Time-resolved photoluminescence lifetime of quasi-2D perovskite films with different PVP concentrations

The photoluminescence (PL) spectra of quasi-2D perovskite films with different compositions of PVP are shown in Fig. 5a together with a photograph of the quasi-2D perovskite films emitting bright green light under 365 nm excitation wavelength as an inset. Besides, the PL emission peak gradually blue shifted from 517 nm for pure PPA_2_(CsPbBr_3_)_2_PbBr_4_ thin film to 512 nm, which is in accordance with the reports that small grain size polycrystalline films have a blue-shifted PL peak compared with big grain polycrystalline film [34]. Meanwhile, the perovskite film with the PVP concentration of 3 mg/mL shows the highest PL intensity under the same excitation condition which can also be proven in the inset photograph. To understand the effect of PVP concentration on the exciton properties of perovskite films, we measured the TRPL of perovskite films as shown in Fig. 5b, which match bi-exponential expression (1) well [35]:

1 $$ I={A}_1{e}^{-\frac{t}{\tau_1}}+{A}_2{e}^{-\frac{t}{\tau_2}} $$

in which *I* represents the normalized PL intensity, *A*_1_ and *A*_2_ stand for the proportion of the components, and *τ*_1_ and *τ*_2_ represent the respective exciton lifetime for different carrier kinetic process. The average lifetime (*τ*_avg_) is calculated in the following expression (2):

2 $$ {\tau}_{\mathrm{avg}}=\frac{A_1{\tau}_1^2+{A}_2{\tau}_2^2}{A_1{\tau}_1+{A}_2{\tau}_2} $$

The PL lifetime of the quasi-2D perovskite is considered as the summation of fast decay and slow decay components, which is characterized by a short lifetime *τ*_1_ and along lifetime *τ*_2_. The fitted values are shown in Table 1. The average time for pure PPA_2_(CsPbBr_3_)_*n*-1_PbBr_4_ is small (7.5 ns), which is improved significantly by introducing PVP as an additive. And with increasing the PVP concentration in the precursor solution, the *τ*_avg_ of 3-mg/mL PVP-based perovskite film shows the largest average lifetime of 19.88 ns, indicating that the defect state density is decreased. When the excess PVP of 4 mg/mL is introduced, the average lifetime of the perovskite film decreases, which may be due to the emerging defect state caused by the rough perovskite film as shown in Fig. 3d. According to the above analysis, we can get the conclusion that the proper PVP in perovskite can decrease the trap density through passive grain boundaries, favorable for the performance of PeLEDs [31].

**Table 1:** Detail-fitted parameters of the time-resolved photoluminescence decay curve

PVP ratio	*A*_1_ (%)	*τ*_1_ (ns)	*A*_2_ (%)	*τ*_2_ (ns)	*τ*_avg_ (ns)
Without PVP	60.58	1.85	39.42	9.24	7.5
2 mg/mL	58.88	3.89	41.12	17.61	14.31
3 mg/mL	49.82	5.57	50.18	23.28	19.88
4 mg/mL	63.06	3.15	36.94	13.25	10.33

**Fig. 6:**
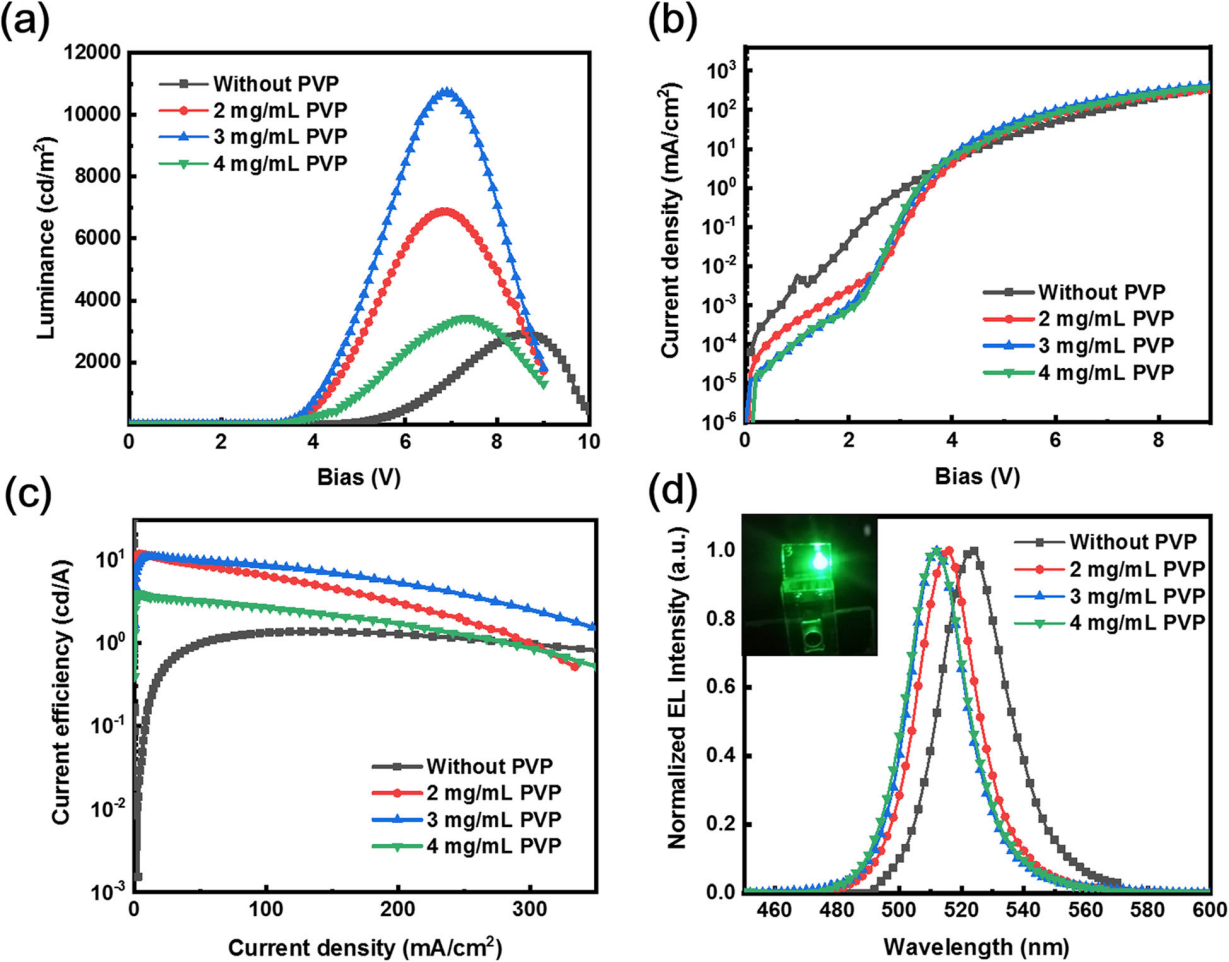
**a** Luminance versus voltage (L-V), **b** current density versus voltage curves (J-V), and **c** current efficiency versus current density (CE-J) characteristic curves of quasi-2D PeLEDs based on different PVP concentrations. **d** Normalized EL spectra of quasi-2D PeLEDs based on different PVP concentrations. Bright EL photography of quasi-2D PeLED with 3 mg/mL PVP is shown in the inset

To explore the availability of PVP incorporation in quasi-2D PeLED, the PeLEDs with different volume ratios of PVP with the same device architecture are displayed in Fig. 1. The luminance-voltage (L-V) and current density-voltage (J-V) curves of quasi-2D PeLEDs with different concentrations of PVP and corresponding CE curves are shown in Fig. 6a–c, respectively. The performance of quasi-2D PeLEDs without and with PVP is summarized in Table 2.

**Table 2:** Summarized performance of quasi-2D PeLEDs with different concentrations of PVP

PVP ratio	*V*_turn-on_ (*V*)	*L*_max_ (cd m^−2^)	CE_max_ (cd A^−1^)	EL peak (nm)	PL peak (nm)
Without PVP	3.5	2920	1.38	523.0	522.4
2 mg/mL	3.2	6870	10.83	516.2	515.4
3 mg/mL	3.1	10,700	11.68	513.0	512.2
4 mg/mL	3.1	3430	3.97	512.0	511.1

The PeLEDs with pure PPA_2_(CsPbBr_3_)_2_PbBr_4_ have a maximum luminance of 2920 cd m^−2^, while the CE is limited to 1.38 cd A^−1^. The reason for this poor performance may be because of the poor film morphology with a series of pinholes and the grain boundary defects. As shown in Fig. 6b, the addition of PVP significantly reduces the leakage current at low voltages, demonstrating that the shunting paths are suppressed in perovskite film. The result matches well with the morphology characterization. The PeLED with 2 mg/mL PVP demonstrates the improved peak brightness of 6870 cd m^−2^, with a CE of 10.83 cd A^−1^ as shown in Fig. 6a, c. When the concentration of PVP is increased, the maximum luminance and CE got further improvements, of which the device with the PVP of 3 mg/mL exhibits the peak luminance of 10,720 cd m^−2^, which is a nearly fivefold improvement compared with that of the device without PVP as an additive, and CE increased to 11.68 cd A^−1^. Besides, the electroluminescence (EL) characteristics of the quasi-2D PeLEDs are tested in Fig. 6d. The EL peaks of PeLED incorporation with different concentrations of PVP show the same trend as the PL peaks of corresponding films. With the increase of the PVP incorporation ratio, the EL peaks blue shift from 522 to 516, 513, and 512 nm. This phenomenon can be concluded that PVP restricts the growth of perovskite grains, resulting in the reduction of grain size and the blue shift of EL peak.

**Fig. 7:**
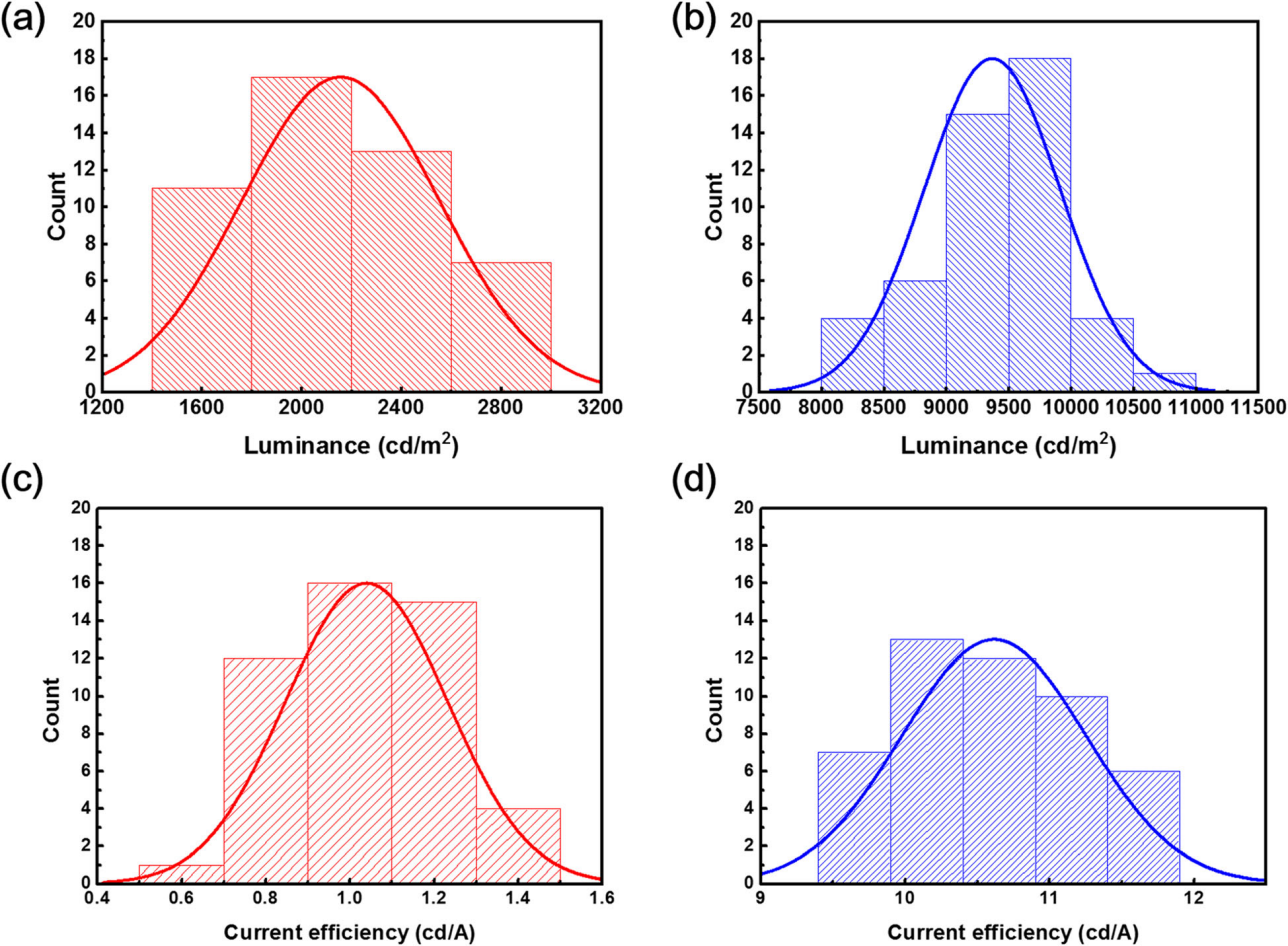
Performance distribution of the quasi-2D PeLEDs. Maximum luminance of quasi-2D PeLED **a** without PVP as an additive and **b** with 3 mg/mL PVP, respectively. Maximum CE of quasi-2D PeLED **c** without PVP as an additive and **d** with 3 mg/mL PVP, respectively

To test the repeatability of our devices, we set up two groups without PVP and with 2 mg/mL PVP treatment. Each group of 48 devices was produced using the same fabrication process. The luminance and CE histograms of PeLEDs with Gaussian fitting are displayed in Fig. 7. The maximum luminance and CE of quasi-2D PeLEDs without PVP (50%) exceed 2200 cd m^−2^ and 1.1 cd A^−1^, respectively, as shown in Fig. 7a, c. However, most of the fabricated PVP-based quasi-2D PeLEDs (60%) yield a maximum luminance and CE of over 9000 cd m^−2^ and 10 cd A^−1^, respectively, as shown in Fig. 7b, d. These results confirm that the PVP additive can improve the performance of the quasi-PeLEDs again, which also proved that PVP-based quasi-2D PeLEDs have better reproducibility than control devices.

## Conclusions

In conclusion, high-performance quasi-2D PeLEDs have been demonstrated with a CE up to 11.68 cd A^−1^ via a polymeric additive of PVP. The result showed that the PVP additive enables the formation of compact, smooth, and pinhole-free perovskite films with a small grain size. The current leakage and non-radiative recombination have been suppressed significantly via PVP treatment. Hence, compared with the poor performance of control devices (without PVP), a substantial increase in both brightness and efficiency has been achieved in quasi-2D PeLEDs with PVP, among which the best device yields a CE of 11.68 cd A^−1^ and maximum luminance of 10,700 cd m^−2^. This method may provide a guide for the morphology control of quasi-2D perovskite films, thus improving the performance of perovskite optoelectronic devices, furthermore.

## Data Availability

All data are fully available without restriction.

## Abbreviations

AFM: Atomic force microscope
Al: Aluminum
BABr: Butylammonium bromide
CE: Current efficiency
CsBr: Cesium bromide
DMSO: Dimethyl sulfoxide
EL: Electroluminescence
EQE: External quantum efficiency
ITO: Indium tin oxide
J-V: Current density-voltage
LiF: Lithium fluoride
L-V: Luminance-voltage
OHIP: Organic-inorganic hybrid perovskites
PABr: Propylammonium bromide
PBABr: Phenylbutylammonium bromide
PbBr_2_: Lead bromide
PEABr: Phenethylammonium bromide
PEDOT:PSS: Poly(3,4-ethylenedioxythiophene):poly(styrene-sulfonate)
PeLEDs: Perovskite light-emitting diodes
PL: Photoluminescence
PLQY: Photoluminescence quantum yield
PPA: Phenylpropylammonium
PVP: Poly(vinylpyrrolidone)
quasi-2D: Ruddlesden-Popper two-dimensional
SEM: Scanning electron microscope
XRD: X-ray diffraction
